# Trauma as a critical condition in the very old: assessment, care pathways and outcome differences

**DOI:** 10.1007/s41999-026-01512-z

**Published:** 2026-06-18

**Authors:** Susannah Leaver, Simon Conroy, Guy Lumley, Kate Hancorn, Elaine Cole

**Affiliations:** 1 Department of Critical Care, St George’s University Hospitals NHS Foundation Trust, London, UK; 2 Older People’s Service, The Royal London Hospital, London, UK; 3 Department of Surgery, The Royal London Hospital, London, UK; 4 Centre for Trauma Sciences, Blizard Institute, Queen Mary University of London, London, UK

**Keywords:** Geriatric trauma, Frailty, Triage, Critical care, Outcomes

## Abstract

Trauma in very old adults represents a growing and distinct form of critical illness, reflecting demographic aging and increasing levels of frailty, multimorbidity, and dependency. Unlike younger populations, where trauma is typically driven by high-energy mechanisms, injury in older adults most commonly results from low-energy falls. Despite this, the consequences are often severe, with high rates of hemorrhage, traumatic brain injury and thoracic trauma, and subsequent multi-organ dysfunction requiring critical care. Age-related physiological changes, combined with multimorbidity and polypharmacy, alter responses to injury and may mask early signs of deterioration. As a result, commonly used triage systems frequently underestimate injury severity in older patients, contributing to under-triage and delays in access to specialist trauma care. Frailty is increasingly recognized as a key determinant of outcomes, influencing survival, length of stay, and functional recovery, and should be routinely assessed early in the patient pathway. Assessment of frailty and intrinsic capacity in the Emergency Department is often challenging, particularly when relatives or caregivers are unavailable to provide collateral history. Older adults should receive an in-depth assessment and documentation of pre-injury status and prior wishes; however, this is inconsistently performed in routine trauma practice. Management of older trauma patients is complex and often fragmented across specialties, with significant variability in care pathways. Decision-making is further complicated by prognostic uncertainty and the need to align interventions with patient values and baseline function. While ICU admission may improve outcomes in selected patients, survival is frequently accompanied by complications, such as delirium, sarcopenia, and long-term functional impairment. Optimizing outcomes requires a coordinated, multidisciplinary approach that integrates trauma and geriatric principles, emphasizing early recognition, tailored resuscitation, comprehensive assessment of frailty, and patient-centered care. Future priorities include improving triage accuracy and injury recognition, standardizing care pathways, and focusing on meaningful recovery in this vulnerable population.

## Key summary points

**Aim:**

To review trauma in the very old as a distinct form of critical illness, focusing on assessment, care pathways and outcomes.

**Findings:**

Among very old adults, trauma is predominantly related to low-energy falls, yet is associated with substantial morbidity and mortality. Frailty, multimorbidity, and polypharmacy influence injury recognition, treatment decisions, and recovery, and current care pathway remain heterogeneous.

**Message:**

Improved outcomes depend on early recognition, routine frailty informed assessment, and integrated multidisciplinary care aligned with patient goals.

## Introduction

Major trauma resulting in significant injury is a leading cause of global mortality and morbidity[1]. Trauma was traditionally thought of as a disease in the first four decades of life, in particular young men involved in motor vehicle collisions. However, the rapid aging of the European population is reshaping the epidemiology of trauma and its impact on hospital and intensive care services. Across Europe, the number of very old adults is increasing steadily, accompanied by a rising prevalence of frailty, multimorbidity, and functional dependency[2–4]. Consequently, trauma in older adults is increasing in both absolute and relative terms and now represents a significant and growing contributor to hospitalization, critical illness, and intensive care unit (ICU) admission in later life [5–7].

In contrast to younger populations, trauma in older adults is usually due to low-energy mechanisms, particularly falls from standing height [6, 8, 9]. Despite their apparently minor nature, such mechanisms can be associated with severe traumatic brain injury, thoracic trauma, hemorrhage, and subsequent organ dysfunction requiring ICU admission [6, 10]. Trauma in older adults, particularly among those who are frail, is associated with prolonged length of stay, functional decline, disability, mortality and new institutionalization [11–14].

Physiological aging fundamentally alters responses to injury and critical illness. Multimorbidity and polypharmacy further complicate early assessment and resuscitation, contributing to atypical presentations and masking physiological deterioration [15, 16]. Triage tools, largely developed and validated in younger cohorts, frequently underestimate injury severity in older adults, leading to under-triage and delayed access to trauma team activation, imaging, and critical care [17] [18].

Once admitted to hospital, the care of older trauma patients is frequently complex and fragmented. Older patients with major trauma may be managed in a wide variety of settings, such as either dedicated trauma units or on geriatric, general surgical, orthopedic, or neurosurgical wards. Decisions regarding surgical intervention, primary treating team, ICU admission, and treatment escalation are often made in the context of prognostic uncertainty, competing comorbidities, and limited assimilation of baseline function and frailty into decision-making [19]. Outcomes following ICU admission are heterogeneous; while survival may be achieved, it is frequently accompanied by delirium, immobility, sarcopenia, multi-organ dysfunction, frailty and long-term functional impairment [12, 20, 21].

This review examines trauma as a distinct and increasingly relevant cause of critical illness in later life. We explore epidemiology and injury mechanisms, age-related physiological changes, challenges in triage and early management, surgical and intensive care considerations, post-ICU outcomes, and opportunities for prevention.

## Epidemiology and injury mechanisms

Large registry and observational data show that the “typical” major trauma patient is getting older [5, 7]. In 2015, the UK national trauma registry reported an almost 20 year increase in the average age of severely injured patients admitted to hospital from 36 to 54 years. This was associated with a marked rise in major trauma seen in people aged ≥ 75 years [5]. Likewise, the mean age of trauma patients rose from 39 to 51 years in the German Trauma Register and the proportion of older trauma patients aged 65 years or over in the National Trauma Database (USA) increased from 18 to 30%[22]. Similarly, the Quebec Trauma Registry in Canada reported a median age increase from 57 to 67 years and there was a 12.3% increase in the proportion of older trauma patients (≥ 65 years) [6].

There has been a marked shift away from high-energy mechanisms such as road traffic collisions toward low-energy trauma, in particular falls from standing height, which now account for a substantial and growing share of major trauma workload [5, 6, 8, 12, 13]. These injuries can have disproportionately severe consequences in older people with associated increases in mortality, resource use and poor longer-term outcome [23, 24]. Multiple datasets have reported an increased severity of injury among older trauma patients [6, 25], which is associated with longer length of acute hospital stay, increased risk of discharge to rehabilitation facilities and mortality [12, 13]. Prolonged immobilization following trauma or falls in older adults may precipitate rhabdomyolysis and acute kidney injury, representing an under-recognized contributor to morbidity and mortality in geriatric trauma populations [26].

This epidemiological transition is accompanied by a distinctive change in injury profile, compared with younger cohorts, where older patients more frequently sustain head and traumatic brain injury (TBI)[27], thoracic injuries (rib fractures/pulmonary contusion)[28, 29], and fragility fractures (hip, pelvic, vertebral) [6, 12, 13, 30]. These injuries can be complicated by pre-injury anticoagulants[31], which are associated with poor outcomes in older TBI populations[32]. Data from 21,681 patients in the CENTER-TBI (Collaborative European NeuroTrauma Effectiveness Research in TBI) Registry revealed that 40% of TBI were caused by low-energy falls, almost half of which were taking pre-injury anti-coagulants and /or platelet aggregation inhibitors[33].

### Frailty and trauma

Frailty reflects an accumulation of health deficits over the life course, resulting in diminished physiological reserve and increased vulnerability to acute stressors [34]. As frailty progresses, seemingly innocuous insults lead to a potentially catastrophic functional decline, slower and incomplete return to baseline. There is growing interest in measuring frailty in the context of trauma [11–14, 35–42]. Increasing frailty is associated with worse outcomes after traumatic injury with a growing recognition of the potential need to adapt services to meet the needs of older people living with frailty [12–14, 36, 37, 39–41].

Frailty is commonly measured using the accumulation of deficits approach—a frailty index (FI) [43]. In clinical practice, frailty should be explicitly identified using validated instruments such as the Clinical Frailty Scale (CFS) [44]. The CFS differs from the FI but ‘taps into’ the same domains, and has the added advantage of being quick, simple and easy to use [45] and a strong predictor of mortality, length of stay, and readmissions in emergency and critical care settings [46]. Assessment must reflect baseline function, typically two weeks prior to presentation, rather than the current state.

#### How does frailty impact clinical assessment and care?

Frailty is often accompanied by multimorbidity, which in turn attracts polypharmacy. Frailty, multimorbidity and polypharmacy can all conspire to precipitate falls in older people (e.g., medication related postural hypotension leading to brain hypoperfusion in the context of small vessel disease). Osteoporosis is often present in the context of frailty and can be related to polypharmacy, such as long-term steroids.

Furthermore, this combination of frailty, multimorbidity, and polypharmacy can have a major influence on anatomy and physiology, that in turn complicates clinical care. For example, aging is associated with increased cardiovascular stiffness, impaired diastolic filling, and reduced chronotropic reserve. Consequently, cardiac output in older adults depends heavily on preload. Age-related pulmonary changes include reduced compliance, diminished ventilatory reserve, and impaired gas exchange. Sarcopenia, a defining feature of frailty, not only increases the risk of falls, but has a major impact on recovery and rehabilitation following trauma. Polypharmacy further confounds these issues, for example beta-blockers limiting a chronotropic response to a physiological challenge. These changes limit compensatory responses during critical illnesses such as trauma.

## Triage in the very old: why we miss severity

### Prehospital triage

Early identification of severe injury in older people can be challenging. Trauma systems use triage protocols to identify patients most at risk of severe injury and to ensure timely transportation to appropriately resourced centers. Under-triage occurs when major trauma is not recognized and severely injured patients are transported to non-trauma centers, necessitating a secondary onwards transfer[47, 48]. Existing prehospital trauma triage protocols generally lack sensitivity and specificity for recognizing severe injury in older people and under-triage in geriatric trauma patients remains a considerable concern[49, 50]. As a result, older trauma patients are more likely to be primarily transported to non-specialist hospitals despite being seriously injured, which may lead to delays in diagnostics and management, and ultimately poor outcome [51, 52].

### ED triage and trauma team activation

Rates of emergency department (ED) under-triage in older trauma patients are consistently higher compared to younger cohorts [53, 54]. In response, some settings have redefined triage criteria based on age [55–57] or physiology thresholds similar to those suggested for prehospital care [58, 59]. These guidelines vary widely, lack standardization and do not prevent under-triage [60], but the majority are associated with improved clinical outcomes in severely injured older patients [54].

Standard trauma team activation criteria, based on higher energy mechanisms and traditional vital sign parameters, may result in delayed injury recognition in older patients. A lower threshold for trauma team activation has been advised for those aged 65 years or more[61]. However, the ‘older old’ may be most disadvantaged, where increases in age are inversely related to trauma team activation[62] and criteria lack sensitivity in identifying severe injury in the very old[52, 63]. Given the atypical presentations in these older cohorts, ED trauma assessment must therefore maintain a high index of suspicion for significant injury[64]. In some ED settings, differing levels of ‘expedited’ or adapted trauma assessments, based on low-energy mechanisms, comorbidities or frailty, are successful in identifying (or ruling out) significant injury, achieving timely imaging, etc. within limited resources [65, 66]. A lower threshold for advanced imaging is recommended by the European Society of Emergency Radiology in this patient cohort, with contrast-enhanced computed tomography where available [67].

## Decision-making in frailty related trauma: what’s different?

**Fig. 1:**
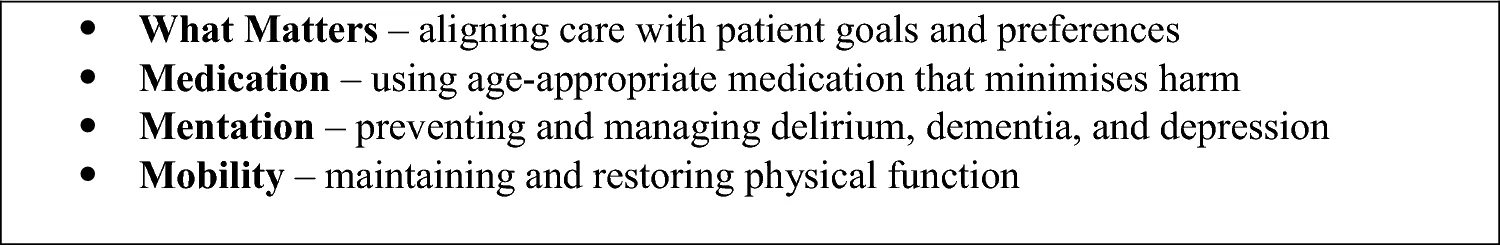
The ‘4Ms’ model: Age friendly care for guiding management in older ICU patients [21]

Sometimes the prognosis is not clear. It can be challenging to hold and communicate the uncertainty that the injured person might die but there remains potential for recovery. An individualized and shared care plan involving the patient, those close to them should be made[68]. Identification of what matters most to the patient is crucial in setting goals of care. This might involve invasive treatment with the primary intention of relief of suffering rather than prolonging life. When death is considered inevitable, focus should be on end of life care. ‘What matters most’ (Fig. 1) might be identified by asking the injured person themselves and actively seeking collateral sources of information such as advanced directives or the input of those close to the patient.

Shared decision-making empowers people to receive care based on their own priorities. A patient’s priorities might differ from the healthcare team, for example based upon family, prior healthcare experiences and faith[69]. One framework to support shared decision-making is BRAN: Benefits, Risks, Alternatives, and what if we do Nothing [70]. Older adults with major traumatic injuries may not have capacity to be involved in shared decision-making because of traumatic injuries, pre-existing cognitive impairment, or delirium. Collateral sources such as advance care plans/powers of attorney or family need to be used to inform decision-making.

The evolution of the patient’s condition over time can give crucial clinical information regarding prognosis and time-limited trials of treatment can be useful to assess patient trajectory[71]. For example, in an older person living with frailty who has reduced consciousness following a fall with a small acute subdural hematoma and co-existent pneumonia, it might be difficult to differentiate the impact of traumatic brain injury and hypoactive delirium precipitated by infection. If aligned with patient-centered goals, a trial of supportive care might provide clarity on the trajectory. Regular clinical review and clear communication is essential.

## In-hospital management

### Resuscitation

World Society of Emergency Surgery (WSES) guidelines note that SBP < 110 mmHg can represent shock and occult hypoperfusion in patients > 65 years and recommends early and serial lactate/base deficit measurement and Point of Care Ultrasound (POCUS) to help monitor cardiac function and fluid status. Admission lactate > 2.4 mmol/L, as a marker of occult hypoperfusion, was associated with markedly higher mortality (34.6% vs 11.6%), supporting lactate as a pragmatic early prognostic marker in these patients[72, 73].

Trauma hemorrhage-related hypothermia, acidosis, hypocalcaemia, and coagulopathy are associated with significant morbidity and mortality [74]. Older patients are at increased risk of developing hypothermia where passive and active warming techniques should be implemented as soon as possible after injury [75]. Trauma major hemorrhage protocols focus on the early recognition and correction of acidosis, hypocalcaemia and coagulopathy[76]. While resuscitation–transfusion priorities do not change with age, the evidence in older patients is limited. A multisite European study reported the same patterns of coagulation dysfunction in younger and older patients, but advanced age was strongly associated with increased fibrinolysis[77]. Tranexamic Acid (TXA) is an antifibrinolytic administered after injury, and in a cohort of more than 10,000 older trauma patients was associated with reduced 24 h mortality without increased risk of thromboembolic events, irrespective of pre‐existing anticoagulation therapy[78].

Early identification of anticoagulation is an important determinant of outcomes in older trauma patients, where hematology-led, anticoagulant reversal protocols should be agreed for those with significant hemorrhage. For emergency reversal of vitamin K antagonists, intravenous vitamin K and 4-factor prothrombin complex concentrate (PCC) are recommended. For DOACs, Idarucizumab may be used to reverse Dabigatran, and Andexanet for Rivaroxaban and Apixaban, with PCC recommended if these are unavailable [73].

Older people are at increased risk of venous thromboembolism (VTE) after injury, but the timing of chemoprophylaxis can be challenging. In those with traumatic brain injury, a recent scoping review suggested initiation within 24–72 h, in the absence of TBI progression, life-threatening bleeding, or requirement for surgery [79]. In patients with solid organ injuries, VTE prophylaxis should be withheld until bleeding and coagulopathy are controlled [80].

The Geriatric Trauma Outcome Score (GTOS) uses age, injury severity score, and blood transfusion within 24 h of hospitalization to predict mortality in older trauma patients although has been criticized for not accounting for frailty or comorbidities[81]. Injury severity scoring is not calculated until all imaging results are available, therefore GTOS may not have utility in the initial hours and days after injury. Similarly, early consideration of frailty and comorbid state is important for outcome prognostication. There is some evidence that early geriatrician input might improve trauma related outcomes; how this can be achieved at scale is an important unanswered research question [82].

### Intensive care management

Trauma patients require ICU for organ support, when severe injuries or physiological instability necessitate advanced monitoring due to high risk of organ failure or rapid deterioration, after severe injuries or following major trauma surgery, and in some cases for complex pain management. In patients aged ≥ 65 years with clinically significant blunt thoracic trauma, ICU admission is associated with improved outcomes compared with ward management, including lower in-hospital mortality and a greater likelihood of discharge home[83].

However, multi-organ dysfunction syndrome (MODS) can develop early after trauma ICU admission and outcomes are worse in older patients[84]. Among patients aged ≥ 65 years, frailty is associated with the development of MODS, and frail patients who develop MODS had higher mortality. Other risk factors include traumatic brain injury (TBI) and greater injury severity [10]. Importantly, most older patients who do not develop MODS during the ICU stay had favorable outcomes, with approximately half returning home.

Evidence relating specifically to very old trauma patients admitted to ICU remains limited. Duncan et al. reported that in patients aged ≥ 80 years admitted to ICU, 30-day mortality was high (54%), and both higher organ failure scores and traumatic brain injury were associated with worse outcomes[11]. Similarly, Legros reported that in trauma patients aged ≥ 85 years admitted to ICU, 30-day mortality was 45.5%, and factors associated with ICU mortality included TBI (GCS < 15), pre-hospital hemoglobin < 13, and severe injury (ISS > 16). Withdrawal of life-sustaining treatment occurred in a quarter of patients, with 21.3% occurring within the first 48 h. In both trials, similar to other ICU trials, age itself was not associated with outcome [11–13, 19]. However, contrary to other trials, frailty measured using the CFS was not associated with 30-day mortality, but this may reflect pre-ICU triage of older trauma populations.

#### Specific management on the ICU for older trauma patients

##### Preventing and managing delirium

Delirium is extremely common in ICU patients, with an incidence of up to 80% in those receiving mechanical ventilation and 20–50% in other ICU patients[85]. Delirium is an independent predictor of prolonged ICU and hospital length of stay, increased mortality, and long-term cognitive impairment. Risk factors include advanced age, frailty, and comorbidity burden, alongside peri-traumatic factors such as infection, hypotension, and hypothermia which further increase susceptibility in older trauma patients[86]. The WSES guidelines recommend an early and systematic assessment for delirium in this population due to its association with unfavorable outcomes[73].

Delirium frequently goes unrecognized in both the ICU and ward setting and should be assessed regularly using validated tools such as the Confusion Assessment Method for the ICU (CAM-ICU). Hypoactive delirium, in particular, may present as withdrawal or reduced responsiveness and can be mistaken for adequate comfort or absence of pain. Routine use of antipsychotic medication for delirium prevention is not recommended, and there remains no clear consensus regarding pharmacological treatment strategies in these patients[87]. Clinicians should therefore prioritize evidence-based non-pharmacological interventions. Implementation of the ABCDEF bundle (See Table 1) in ICU has been associated with improved survival, reduced duration of delirium, reduced ICU readmission, and an increased likelihood of discharge home[88]. Additional strategies, such as minimizing sleep disruption, ensuring use of sensory aids, and promoting orientation, should be routinely implemented [21].

**Table 1:** ABCDEF bundle for the prevention and management of delirium [87]

A	Assess, prevent, and manage pain
B	Both spontaneous awakening and breathing trials
C	Choice of analgesia and sedation
D	Delirium: assess, prevent, and manage
E	Early mobility and exercise
F	Family engagement and empowerment

##### Pain management

Effective pain management is a critical and often under-recognized component of delirium prevention and treatment. In older trauma patients, a multimodal, opioid-sparing analgesic regimen is recommended [73]. This includes regular paracetamol, lidocaine patches, gabapentinoids, and where appropriate NSAIDs for severe pain, with careful consideration of potential adverse effects. Opioids and short acting opiates may be used for breakthrough pain but should be carefully titrated to clinical response. A recent meta-analysis demonstrated a near doubling of delirium in older inpatients experiencing pain[89], highlighting the importance of adequate analgesia. Regional anesthesia should be used whenever possible[90]. Techniques, such as peripheral nerve blocks for hip fractures and serratus anterior or paravertebral blocks for rib fractures [73], reduce opioid requirements and are associated with lower rates of delirium and respiratory complications and enable earlier mobilization. In a multicenter study of older ICU patients with three or more rib fractures, thoracic regional analgesia was associated with a 35% reduction in delirium [91].

##### Early mobilization

Early mobilization and rehabilitation are critical in older ICU patients as it aids prevention of ICU acquired weakness, improves functional outcomes and helps prevent and treat delirium. A landmark randomized controlled trial demonstrated that in mechanically ventilated ICU patients, a daily sedation interruption combined with early physiotherapy and occupational therapy improved return to independent functional status at hospital discharge (59% vs. 35%), reduced delirium duration, increased ventilator-free days, and was safe[92]. Consequently, effective pain control together with early mobilization is recommended in older ICU patients [21, 87].

### Trauma surgery in older patients

Operating on older adults presents several important differences across physical, anatomical, and clinical domains. An awareness of these challenges allows the surgeon to alter approach, decision-making and clinical care to reduce complications and identify them early should they be encountered.

For the surgeon, age-related nuances in decision-making present part of the challenge for older trauma patients. Permissive hypotension is a key principle of damage control resuscitation to reduce blood loss, but is likely less well tolerated in older patients and must be utilized judiciously[67]. Increasing age is an independent predictor of poor outcome after surgery[93]. Older patients have reduced physiological reserve and therefore the risk associated with operative intervention may be higher in this cohort[94].

When deciding treatment between surgery or interventional radiology or conservative management, a less-aggressive management plan may warrant more thought than in younger patients due to increased complexity and complication risk. The impact of fragile tissues and reduced vessel elasticity can make surgical procedures more challenging. This, together with slower wound healing and increased infection risk [95] may advocate for more conservative management in some older patients. To reduce risks of re-bleeding in splenic trauma, interventional radiology (IR) may be considered as an alternative to surgery and splenectomy, which would be preferable in younger populations[96]. However, with reduced physiological reserve and limited ability to compensate, an aggressive surgical treatment is better indicated in the early phases of trauma hemorrhage to improve outcome[64]. The risk of complications in the post-operative period mandates close vigilance. Background nutritional status, which influences surgical outcome and complication rate, is poorly measured and documented in older patients and represents an opportunity for future trauma research.

### Rehabilitation and recovery

**Fig. 2:**
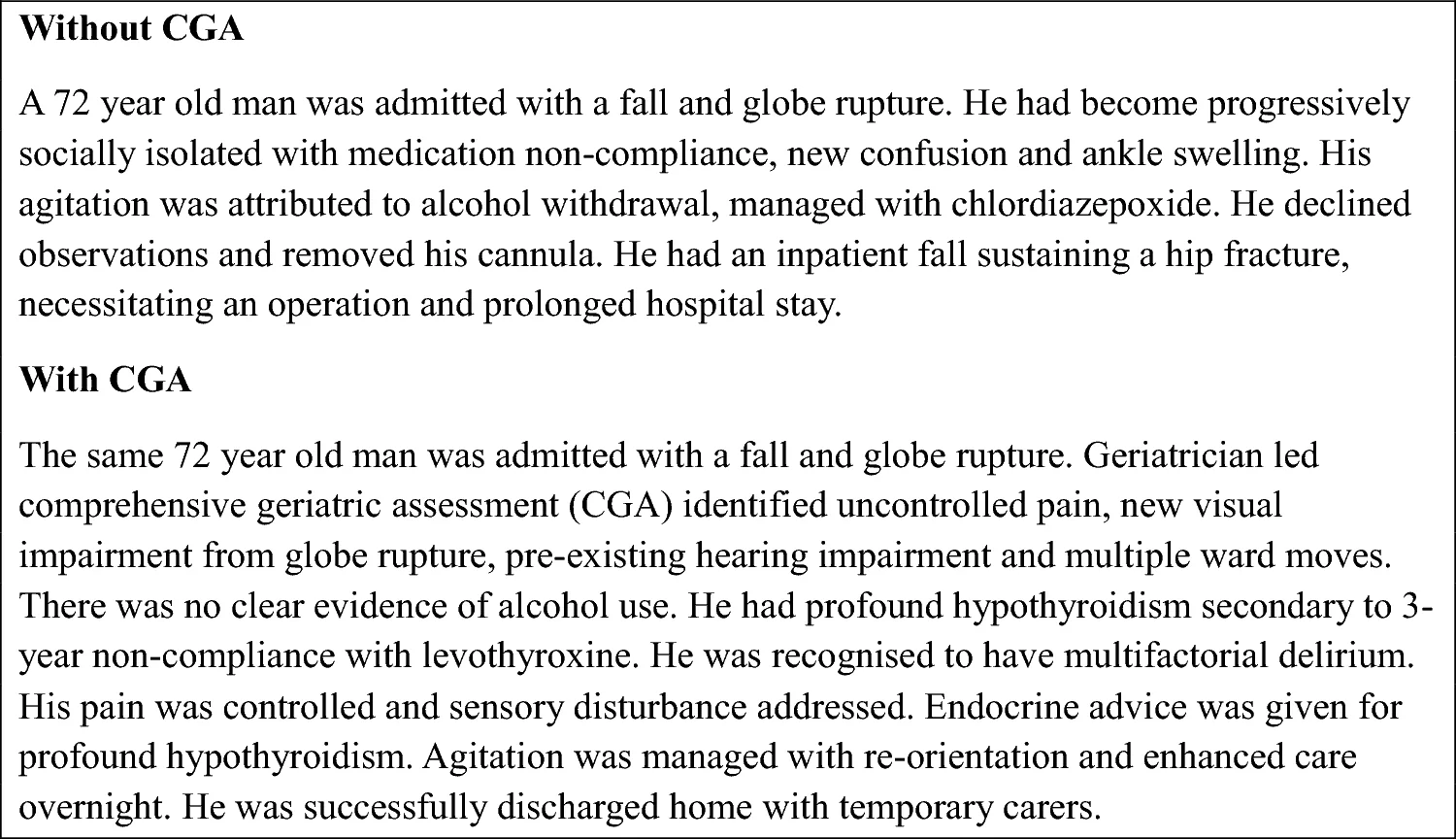
Case vignette—care with and without a holistic assessment

The growing prevalence of frailty and disability among older trauma patients requires aging-focused care that extends beyond survival and post-ICU trajectories to consider functional recovery and outcomes most important to patients[21]. Rehabilitation therapists should complete a personalized and holistic needs assessment in partnership with the patient and family or carers as soon as possible after injury[97]. A major goal of trauma rehabilitation is to prevent the secondary harms of injury, particularly deconditioning syndrome. People living with frailty are at increased risk of further functional impairment after injury because of prolonged bed rest[98]. It is thus important to quickly identify and manage barriers to early mobilization e.g., pain and movement restrictions, which might relate to concerns about fracture stability, where there are serious risks of spinal compromise potentially leading to irreversible harm. However, there is increasing recognition that immobilization comes with harms that need to be balanced[99]. For example, cervical collars may affect swallowing, increase delirium, lead to pressure ulcers and impact upon length of stay and discharge destination[100]. Parallels can be drawn with the benefits of early surgery for hip fractures in older adults; minimizing secondary harm by prioritizing benefits of early mobilization, which also results in reduced length of stay and reduced costs [101]. Shared decision-making can support balancing risks in the context of individualized patient recovery goals, and this may be best met through Comprehensive Geriatric Assessment (CGA) or trauma–geriatrician involvement[102] (Fig. 2). Finally, traumatic injury in the very old offers important opportunities for secondary and tertiary prevention, particularly through falls prevention, optimization of bone health, medication reviews, and future care planning.

## Future directions and research priorities (EC/SC)

The increasing number of older people sustaining severe injury means that there are significant research opportunities. Large, multisite studies are required to identify optimal patient management pathways and to better understand factors associated with improved outcomes. Several research priorities have been published [103–105], which focus on but are not limited to:

What are the optimal pre-hospital risk assessment and triage assessments for older injured people?

What is the role and benefits of geriatricians and frailty experts in trauma pathways?

Which outcomes matter most to older trauma patients?

## Conclusions

Trauma in the very old should be regarded as a distinct and increasingly common form of critical illness, characterized by low-energy mechanisms, atypical presentation, under-recognition of severity, and outcomes that are shaped by frailty, multimorbidity, and pre-injury function. Along the patient pathway, from prehospital triage and emergency assessment to surgery, intensive care, ward care, and rehabilitation, older trauma patients require an approach that is frailty-attuned, multidisciplinary, and goal-directed toward what matters most.

Early recognition of occult injury and physiological compromise, routine assessment of frailty and delirium, careful attention to analgesia, early mobilization/rehabilitation, and integration of geriatric expertise are all central to improving outcomes. Assessment of frailty and baseline functional status is often difficult at ED presentation, particularly in the absence of collateral history, underscoring the importance of routine documentation of functional status in older adults. Although mortality remains high in the oldest and frailest patients, many survive hospitalization, and meaningful recovery is possible when care is aligned with baseline function, patient priorities, and realistic treatment goals.

Future trauma systems must therefore move beyond a purely injury-centered model toward one that combines trauma expertise with geriatric principles, in order to reduce avoidable harm, support recovery, and improve both survival and longer-term functional outcomes in this growing population.

## References

[1] WHO. Injuries and violence. 2021 https://www.who.int/news-room/fact-sheets/detail/injuries-and-violence

[2] Collaborators GBDD (2025) Global age-sex-specific all-cause mortality and life expectancy estimates for 204 countries and territories and 660 subnational locations, 1950-2023: a demographic analysis for the Global Burden of Disease Study 2023. Lancet 406(10513):1731–1810 41092927 10.1016/S0140-6736(25)01330-3 PMC12535839

[3] Carrasco-Ribelles LA, Roso-Llorach A, Cabrera-Bean M, Costa-Garrido A, Zabaleta-Del-Olmo E, Toran-Monserrat P et al (2022) Dynamics of multimorbidity and frailty, and their contribution to mortality, nursing home and home care need: a primary care cohort of 1 456 052 ageing people. EClinicalMedicine 52:101610 36034409 10.1016/j.eclinm.2022.101610 PMC9399153

[4] Börsch-Supan A, Brandt M, Hunkler C, Kneip T, Korbmacher J, Malter F et al (2013) Data resource profile: the Survey of Health, Ageing and Retirement in Europe (SHARE). Int J Epidemiol 42(4):992–1001 23778574 10.1093/ije/dyt088 PMC3780997

[5] Kehoe A, Smith JE, Edwards A, Yates D, Lecky F (2015) The changing face of major trauma in the UK. Emerg Med J 32(12):911–915 26598629 10.1136/emermed-2015-205265 PMC4717354

[6] Benhamed A, Batomen B, Boucher V, Yadav K, Mercier E, Isaac CJ et al (2023) Epidemiology, injury pattern and outcome of older trauma patients: A 15-year study of level-I trauma centers. PLoS ONE 18(1):e0280345 36716316 10.1371/journal.pone.0280345 PMC9886263

[7] Dinh MM, Roncal S, Byrne CM, Petchell J (2013) Growing trend in older patients with severe injuries: mortality and mechanisms of injury between 1991 and 2010 at an inner city major trauma centre. ANZ J Surg 83(1–2):65–69 22882777 10.1111/j.1445-2197.2012.06180.x

[8] Dixon JR, Lecky F, Bouamra O, Dixon P, Wilson F, Edwards A et al (2020) Age and the distribution of major injury across a national trauma system. Age Ageing 49(2):218–226 31763677 10.1093/ageing/afz151 PMC7047820

[9] Chen Y, Dai F, Huang S, Qi D, Peng C, Zhang A et al (2025) Global, regional, and national burden of falls among older adults: findings from the Global Burden of Disease Study 2021 and projections to 2040. NPJ Aging 11(1):85 41068164 10.1038/s41514-025-00275-4 PMC12511421

[10] Cole E, Aylwin C, Christie R, Dillane B, Farrah H, Hopkins P et al (2022) Multiple Organ Dysfunction in Older Major Trauma Critical Care Patients: A Multicenter Prospective Observational Study. Ann Surg Open 3(2):e174 36936724 10.1097/AS9.0000000000000174 PMC10013163

[11] Duncan CF, Lonsdale DO, Farrah H, Farnell-Ward S, Ryan C, Watson X et al (2024) 30-Day mortality among very old patients admitted to European intensive care units for major trauma. Gerontology 70(7):715–723 38387455 10.1159/000537718

[12] Legros V, Seube-Remy PA, Floch T, Chauchard C, Leclercq-Rouget M, Prevot-Minella PA et al (2024) Frailty and 6-month trajectory of elderly trauma patients over the age of 65 years admitted to intensive care unit for severe trauma: experience of a level 1 trauma center. BMC Geriatr 24(1):759 39277744 10.1186/s12877-024-05350-1 PMC11401312

[13] Legros V, Picard B, Pasqueron J, Kanagaratnam L, Garrigue D, Rozenberg E et al (2024) Prognosis of major trauma in patients older than 85 years admitted to the ICU, a registry-based study. Eur J Trauma Emerg Surg 50(6):3199–3208 39078492 10.1007/s00068-024-02622-8

[14] Carter B, Short R, Bouamra O, Parry F, Shipway D, Thompson J et al (2022) A national study of 23 major trauma centres to investigate the effect of frailty on clinical outcomes in older people admitted with serious injury in England (FiTR 1): a multicentre observational study. Lancet Healthy Longev 3(8):e540–e548 36102763 10.1016/S2666-7568(22)00122-2

[15] de Groot B, Stolwijk F, Warmerdam M, Lucke JA, Singh GK, Abbas M et al (2017) The most commonly used disease severity scores are inappropriate for risk stratification of older emergency department sepsis patients: an observational multi-centre study. Scand J Trauma Resusc Emerg Med 25(1):91 28893325 10.1186/s13049-017-0436-3 PMC5594503

[16] Nemec M, Koller M, Nickel C (2010) Patients presenting to the emergency department with non-specific complaints: the Basel non-specific complaints (BANC) study. Acad Emerg Med 17(3):284–292 20370761 10.1111/j.1553-2712.2009.00658.x

[17] Alshibani A, Alharbi M, Conroy S (2021) Under-triage of older trauma patients in prehospital care: a systematic review. Eur Geriatr Med 12(5):903–919 34110604 10.1007/s41999-021-00512-5 PMC8463357

[18] Kishawi SK, Mahajan A, Nahmias J, Petrone P, Ho VP, Group ESM (2025) Patterns and implications of missed injuries on computed tomography imaging in older blunt trauma patients. Surgery 185:109524 40639281 10.1016/j.surg.2025.109524

[19] Flaatten H, De Lange DW, Morandi A, Andersen FH, Artigas A, Bertolini G et al (2017) The impact of frailty on ICU and 30-day mortality and the level of care in very elderly patients (>/= 80 years). Intensive Care Med 43(12):1820–1828 28936626 10.1007/s00134-017-4940-8

[20] Brummel NE, Girard TD, Pandharipande PP, Thompson JL, Jarrett RT, Raman R et al (2020) Prevalence and course of frailty in survivors of critical illness. Crit Care Med 48(10):1419–1426 32618688 10.1097/CCM.0000000000004444 PMC7941759

[21] Ferrante LE, Vallet H, Ho JQ, Brummel NE (2026) Challenges and strategies in the care of older adults across the continuum of intensive and post-intensive care medicine. Intensive Care Med 52(1):63–75 41428207 10.1007/s00134-025-08253-w

[22] Jiang L, Zheng Z, Zhang M (2020) The incidence of geriatric trauma is increasing and comparison of different scoring tools for the prediction of in-hospital mortality in geriatric trauma patients. World J Emerg Surg 15(1):59 33076958 10.1186/s13017-020-00340-1 PMC7574576

[23] Cuevas-Østrem M, Røise O, Wisborg T, Jeppesen E (2021) Epidemiology of geriatric trauma patients in Norway: a nationwide analysis of Norwegian Trauma Registry data, 2015-2018. A retrospective cohort study. Injury 52(3):450–459 33243523 10.1016/j.injury.2020.11.007

[24] Cole E, Crouch R, Baxter M, Wang C, Sivapathasuntharam D, Peck G et al (2024) Investigating the effects of frailty on six-month outcomes in older trauma patients admitted to UK major trauma centres: a multi-centre follow up study. Scand J Trauma Resusc Emerg Med 32(1):1 38178162 10.1186/s13049-023-01169-8 PMC10768225

[25] TARN (2017) Major trauma in older people. The Trauma Audit and Research Network

[26] Wongrakpanich S, Kallis C, Prasad P, Rangaswami J, Rosenzweig A (2018) The study of rhabdomyolysis in the elderly: an epidemiological study and single center experience. Aging Dis 9(1):1–7 29392076 10.14336/AD.2017.0304 PMC5772847

[27] Nunes N, Nasef H, Baum S, Kumar S, Werling A, Patel H et al (2026) Outcomes of adult & geriatric trauma patients with severe traumatic brain injury treated at level I or II ACS-verified trauma centers: towards optimizing geriatric trauma care. Am J Emerg Med 101:61–70 41468620 10.1016/j.ajem.2025.12.027

[28] Battle C, Carter K, Newey L, Giamello JD, Melchio R, Hutchings H (2023) Risk factors that predict mortality in patients with blunt chest wall trauma: an updated systematic review and meta-analysis. Emerg Med J 40(5):369–378 36241371 10.1136/emermed-2021-212184

[29] Ferrah N, Cameron P, Gabbe B, Fitzgerald M, Judson R, Marasco S et al (2019) Ageing population has changed the nature of major thoracic injury. Emerg Med J 36(6):340–345 30940714 10.1136/emermed-2018-207943

[30] Ferrah N, Dipnall J, Gabbe B, Cameron P, Ibrahim J, Beck B (2022) Injury profiles and clinical management of older patients with major trauma. Australas J Ageing 41(1):116–125 34611973 10.1111/ajag.13000

[31] Afzal S, Zaidi STR, Merchant HA, Babar ZU, Hasan SS (2021) Prescribing trends of oral anticoagulants in England over the last decade: a focus on new and old drugs and adverse events reporting. J Thromb Thrombolysis 52(2):646–653 33666824 10.1007/s11239-021-02416-4 PMC7933373

[32] Karamian A, Seifi A, Karamian A, Lucke-Wold B (2024) Incidence of intracranial bleeding in mild traumatic brain injury patients taking oral anticoagulants: a systematic review and meta-analysis. J Neurol 271(7):3849–3868 38755424 10.1007/s00415-024-12424-y

[33] Maas AI, Menon DK, Steyerberg EW, Citerio G, Lecky F, Manley GT et al (2015) Collaborative European NeuroTrauma Effectiveness Research in Traumatic Brain Injury (CENTER-TBI): a prospective longitudinal observational study. Neurosurgery 76(1):67–80 25525693 10.1227/NEU.0000000000000575

[34] Kim DH, Rockwood K (2024) Frailty in older adults. N Engl J Med 391(6):538–548 39115063 10.1056/NEJMra2301292 PMC11634188

[35] Galet C, Bloeser C, Engelbart J, Eyck PT, Torner J, Skeete D. Frailty is associated with poor outcomes in midlife trauma patients. The American Journal of Surgery. 2025;241. 10.1016/j.amjsurg.2024.116157 PMC11821431 39729966

[36] Hamdioglu E, Altuntas M, Celik A, Yavasi O (2025) Predicting mortality and intensive care needs in geriatric trauma patients: a ROC analysis of frailty and trauma scoring systems. Eur J Trauma Emerg Surg 51(1):262 40691669 10.1007/s00068-025-02924-5

[37] Chu H, Chen L, Li J, Li J, Yang D, Yang M et al (2024) Impact of Frailty on Inpatient Outcomes of Acute Traumatic Spinal Cord Injury: Evidence From US National Inpatient Sample. Neurologist 29(2):82–90 37839086 10.1097/NRL.0000000000000532

[38] Yeh TS, Kang JH, Littlejohns TJ, Wu CC, Chen JH, Piravej K et al (2024) Frailty and other factors associated with early outcomes in middle-to older age trauma patients: a prospective cohort study. Am J Geriatr Psychiatry 32(2):244–255 37770348 10.1016/j.jagp.2023.08.016

[39] Rafaqat W, Panossian VS, Abiad M, Ghaddar K, Ilkhani S, Grobman B et al (2024) The impact of frailty on long-term functional outcomes in severely injured geriatric patients. Surgery 176(4):1148–1154 39107141 10.1016/j.surg.2024.06.036

[40] Ibitoye S, Bridgeman-Rutledge L, Short R, Braude P, Pocock L, Carter B. Frailty is associated with long-term outcomes in older trauma patients: A prospective cohort study. Injury. 2024;55(2). 10.1016/j.injury.2023.111265 38101198

[41] Braude P, Carter B, Parry F, Ibitoye S, Rickard F, Walton B et al (2022) Predicting 1 year mortality after traumatic injury using the Clinical Frailty Scale. J Am Geriatr Soc 70(1):158–167 34624144 10.1111/jgs.17472

[42] Alshibani A, Banerjee J, Lecky F, Coats TJ, Alharbi M, Conroy S (2021) New horizons in understanding appropriate prehospital identification and trauma triage for older adults. Open Access Emerg Med 13:117–135 33814934 10.2147/OAEM.S297850 PMC8009532

[43] Rockwood K, Mitnitski A (2007) Frailty in relation to the accumulation of deficits. J Gerontol A Biol Sci Med Sci 62(7):722–727 17634318 10.1093/gerona/62.7.722

[44] Rockwood K, Song X, MacKnight C, Bergman H, Hogan DB, McDowell I et al (2005) A global clinical measure of fitness and frailty in elderly people. CMAJ Canadian Medical Association Journal. 173(5):489–495 16129869 10.1503/cmaj.050051 PMC1188185

[45] Elliott A, Hull L, Conroy S (2017) Frailty identification in the emergency department-a systematic review focussing on feasibility. Age Ageing, pp 1–5 10.1093/ageing/afx019 28200012

[46] Elliott A, Taub N, Banerjee J, Aijaz F, Jones W, Teece L, et al (2020) Does the clinical frailty scale at triage predict outcomes from emergency care for older people? Ann Emerg Med 10.1016/j.annemergmed.2020.09.006 33328147

[47] Boulton AJ, Peel D, Rahman U, Cole E (2021) Evaluation of elderly specific pre-hospital trauma triage criteria: a systematic review. Scand J Trauma Resusc Emerg Med 29(1):127 34461976 10.1186/s13049-021-00940-z PMC8404299

[48] Tillmann BW, Nathens AB, Guttman MP, Freedman C, Pequeno P, Scales DC et al (2026) Triage accuracy and the 2015 Field Trauma Triage Criteria Update. JAMA Netw Open 9(1):e2552092 41490108 10.1001/jamanetworkopen.2025.52092 PMC12771241

[49] Haussner WK, Breyre AM, Bascombe K, Barrett WJ, Camacho MA, Overton-Harris P, et al. Prehospital trauma compendium: management of geriatric trauma patients—a position statement and resource document of NAEMSP. Prehospital emergency care : official journal of the National Association of EMS Physicians and the National Association of State EMS Directors. 2025:1–10.

[50] Fuller G, Pandor A, Essat M, Sabir L, Buckley-Woods H, Chatha H et al (2021) Diagnostic accuracy of prehospital triage tools for identifying major trauma in elderly injured patients: a systematic review. J Trauma Acute Care Surg 90(2):403–412 33502151 10.1097/TA.0000000000003039

[51] Horst MA, Morgan ME, Vernon TM, Bradburn EH, Cook AD, Shtayyeh T et al (2020) The geriatric trauma patient: a neglected individual in a mature trauma system. J Trauma Acute Care Surgery 89(1):192–198 10.1097/TA.0000000000002646 32118822

[52] Koch DA, Becker L, Schweigkofler U, Hagebusch P, Faul P, Waydhas C et al (2025) Undertriage in geriatric trauma: insights from a multicentre cohort study. Scand J Trauma Resusc Emerg Med 33(1):123 40640930 10.1186/s13049-025-01432-0 PMC12247425

[53] Fakhry SM, Shen Y, Wilson NY, Orlando A (2025) Multicenter study of trauma activation criteria and time-sensitive care in older versus younger adults. Trauma Surg Acute Care Open 10(4):e001770 41262856 10.1136/tsaco-2025-001770 PMC12625935

[54] Yang AR, Lundy M, Otaibi B, Johnson KG, Roberts HS, Mahankali P et al (2025) Triage of the injured older adult: a narrative review of nationwide hospital practices. Am J Surg 249:116615 40987141 10.1016/j.amjsurg.2025.116615

[55] Bardes JM, Benjamin E, Schellenberg M, Inaba K, Demetriades D (2019) Old age with a traumatic mechanism of injury should be a trauma team activation criterion. J Emerg Med 57(2):151–155 31078345 10.1016/j.jemermed.2019.04.003

[56] Bérubé M, Pasquotti T, Klassen B, Brisson A, Tze N, Moore L (2020) Implementation of the best practice guidelines on geriatric trauma care: a Canadian perspective. Age Ageing 49(2):227–232 31790137 10.1093/ageing/afz153

[57] Hatton GE, McNutt MK, Cotton BA, Hudson JA, Wade CE, Kao LS (2020) Age-dependent association of occult hypoperfusion and outcomes in trauma. J Am Coll Surg 230(4):417–425 31954820 10.1016/j.jamcollsurg.2019.12.011 PMC7719288

[58] Brown JB, Gestring ML, Forsythe RM, Stassen NA, Billiar TR, Peitzman AB et al (2015) Systolic blood pressure criteria in the National Trauma Triage Protocol for geriatric trauma: 110 is the new 90. J Trauma Acute Care Surg 78(2):352–359 25757122 10.1097/TA.0000000000000523 PMC4620031

[59] Caterino JM, Brown NV, Hamilton MW, Ichwan B, Khaliqdina S, Evans DC et al (2016) Effect of geriatric-specific trauma triage criteria on outcomes in injured older adults: a statewide retrospective cohort study. J Am Geriatr Soc 64(10):1944–1951 27696350 10.1111/jgs.14376 PMC5117430

[60] Anantha RV, Painter MD, Diaz-Garelli F, Nunn AM, Miller PR 3rd, Chang MC et al (2021) Undertriage despite use of geriatric-specific trauma team activation guidelines: who are we missing? Am Surg 87(3):419–426 33026234 10.1177/0003134820951450

[61] Calland JF, Ingraham AM, Martin N, Marshall GT, Schulman CI, Stapleton T et al (2012) Evaluation and management of geriatric trauma: an Eastern Association for the Surgery of Trauma practice management guideline. J Trauma Acute Care Surgery 73(5 Suppl 4):S345–S350 10.1097/TA.0b013e318270191f 23114492

[62] Ringen AH, Gaski IA, Rustad H, Skaga NO, Gaarder C, Naess PA (2019) Improvement in geriatric trauma outcomes in an evolving trauma system. Trauma Surg Acute Care Open 4(1):e000282 31245616 10.1136/tsaco-2018-000282 PMC6560476

[63] Hung KK, Yeung JHH, Cheung CSK, Leung LY, Cheng RCH, Cheung NK et al (2019) Trauma team activation criteria and outcomes of geriatric trauma: 10 year single centre cohort study. Am J Emerg Med 37(3):450–456 30041911 10.1016/j.ajem.2018.06.011

[64] ACS. Best practices guidelines for geriatric trauma 2023 [Available from: https://www.facs.org/media/ubyj2ubl/best-practices-guidelines-geriatric-trauma.pdf.

[65] Sober S, Langman L, Mukhi A, Martin J, Vosswinkel J, Singer AJ et al (2025) Geriatric trauma activation. J Surg Res 315:231–240 41056844 10.1016/j.jss.2025.09.010

[66] Fernandez FB, Ong A, Martin AP, Schwab CW, Wasser T, Butts CA et al (2019) Success of an expedited emergency department triage evaluation system for geriatric trauma patients not meeting trauma activation criteria. Open Access Emerg Med 11:241–247 31754315 10.2147/OAEM.S212617 PMC6825467

[67] Atinga A, Shekkeris A, Fertleman M, Batrick N, Kashef E, Dick E (2018) Trauma in the elderly patient. Br J Radiol 91(1087):20170739 29509505 10.1259/bjr.20170739 PMC6221775

[68] LMTS. Palliative and End of Life Care considerations for older trauma patients 2024 [Available from: https://www.c4ts.qmul.ac.uk/downloads/lmtspalliative---eol-care-in-older-trauma-patients-v1-jan-2024.pdf.

[69] Khashaba AA, Brett SJ, Warner BE. Cultural and religious influences on shared decision-making in treatment escalation planning with older adults in the acute setting: a qualitative secondary analysis of interview studies seeking clinician and patient perspectives. Age and Ageing. 2025;54(12). 10.1093/ageing/afaf357 PMC12708056 41403040

[70] NICE. About shared decision making 2026 [Available from: https://www.nice.org.uk/what-nice-does/our-guidance/about-nice-guidelines/about-shared-decision-making.

[71] Guidet B, de Lange DW, Boumendil A, Leaver S, Watson X, Boulanger C et al (2020) The contribution of frailty, cognition, activity of daily life and comorbidities on outcome in acutely admitted patients over 80 years in European ICUs: the VIP2 study. Intensive Care Med 46(1):57–69 31784798 10.1007/s00134-019-05853-1 PMC7223711

[72] Schulman AM, Claridge JA, Young JS (2002) Young versus old: factors affecting mortality after blunt traumatic injury. Am Surg 68(11):942–947 (**discussion 7-8**) 12455785

[73] De Simone B, Chouillard E, Podda M, Pararas N, de Carvalho DG, Fugazzola P et al (2024) The 2023 WSES guidelines on the management of trauma in elderly and frail patients. World Journal of Emergency Surgery : WJES 19(1):18 38816766 10.1186/s13017-024-00537-8 PMC11140935

[74] Mills JD (2024) Trauma diamond of death: adding calcium to the lethal triad. J Emerg Nurs 50(3):330–335 38705704 10.1016/j.jen.2023.12.011

[75] Snijders BMG, Roos MJ, Keijsers C (2023) Incidences of underlying causes of hypothermia in older patients in the emergency department: a systematic review. Eur Geriatr Med 14(3):411–420 37191873 10.1007/s41999-023-00791-0 PMC10261225

[76] Lammers DT, Holcomb JB (2023) Damage control resuscitation in adult trauma patients: what you need to know. J Trauma Acute Care Surg 95(4):464–471 37735778 10.1097/TA.0000000000004103

[77] Curry NS, Davenport R, Wong H, Gaarder C, Johansson P, Juffermans NP et al (2023) Traumatic coagulopathy in the older patient: analysis of coagulation profiles from the Activation of Coagulation and Inflammation in Trauma-2 (ACIT-2) observational, multicenter study. J Thromb Haemost 21(2):215–226 36700506 10.1016/j.jtha.2022.11.005

[78] Fitschen-Oestern S, Franke GM, Kirsten N, Lefering R, Lippross S, Schröder O et al (2024) Does tranexamic acid have a positive effect on the outcome of older multiple trauma patients on antithrombotic drugs? An analysis using the TraumaRegister DGU(®). Front Med Lausanne 11:1324073 38444412 10.3389/fmed.2024.1324073 PMC10912612

[79] Keirsey M, Niziolek GM (2025) Management of post-injury anticoagulation in the traumatic brain injury patient: a scoping review. Injury 56(2):112159 39799871 10.1016/j.injury.2025.112159

[80] Ley EJ, Brown CVR, Moore EE, Sava JA, Peck K, Ciesla DJ et al (2020) Updated guidelines to reduce venous thromboembolism in trauma patients: a Western Trauma Association critical decisions algorithm. J Trauma Acute Care Surg 89(5):971–981 32590563 10.1097/TA.0000000000002830 PMC7587238

[81] Wang C, Tian S, Wang C (2025) Review of trauma scoring systems in geriatric patients. Medicine (Baltimore) 104(25):e42783 40550066 10.1097/MD.0000000000042783 PMC12187255

[82] Braude P, Short R, Bouamra O, Shipway D, Lecky F, Carlton E et al (2022) A national study of 23 major trauma centres to investigate the effect of a geriatrician assessment on clinical outcomes in older people admitted with serious injury in England (FiTR 2): a multicentre observational cohort study. Lancet Healthy Longev 3(8):e549–e557 36102764 10.1016/S2666-7568(22)00144-1

[83] Pyke OJ Jr., Rubano JA, Vosswinkel JA, McCormack JE, Huang EC, Jawa RS (2017) Admission of elderly blunt thoracic trauma patients directly to the intensive care unit improves outcomes. J Surg Res 219:334–340 29078902 10.1016/j.jss.2017.06.054

[84] Vanzant EL, Hilton RE, Lopez CM, Zhang J, Ungaro RF, Gentile LF et al (2015) Advanced age is associated with worsened outcomes and a unique genomic response in severely injured patients with hemorrhagic shock. Critical Care (London, England) 19:77 25880307 10.1186/s13054-015-0788-x PMC4404112

[85] Stollings JL, Kotfis K, Chanques G, Pun BT, Pandharipande PP, Ely EW (2021) Delirium in critical illness: clinical manifestations, outcomes, and management. Intensive Care Med 47(10):1089–1103 34401939 10.1007/s00134-021-06503-1 PMC8366492

[86] Ormseth CH, LaHue SC, Oldham MA, Josephson SA, Whitaker E, Douglas VC (2023) Predisposing and precipitating factors associated with delirium: a systematic review. JAMA Netw Open 6(1):e2249950 36607634 10.1001/jamanetworkopen.2022.49950 PMC9856673

[87] Lewis K, Balas MC, Stollings JL, McNett M, Girard TD, Chanques G et al (2025) A focused update to the clinical practice guidelines for the prevention and management of pain, anxiety, agitation/sedation, delirium, immobility, and sleep disruption in adult patients in the ICU. Crit Care Med 53(3):e711–e727 39982143 10.1097/CCM.0000000000006574

[88] Pun BT, Balas MC, Barnes-Daly MA, Thompson JL, Aldrich JM, Barr J et al (2019) Caring for critically ill patients with the ABCDEF bundle: results of the ICU liberation collaborative in over 15,000 adults. Crit Care Med 47(1):3–14 30339549 10.1097/CCM.0000000000003482 PMC6298815

[89] White N, Bazo-Alvarez JC, Koopmans M, West E, Sampson EL. Understanding the association between pain and delirium in older hospital inpatients: systematic review and meta-analysis. Age and Ageing. 2024;53(4). 10.1093/ageing/afae073 PMC11014791 38610062

[90] Williams A, Bigham C, Marchbank A (2020) Anaesthetic and surgical management of rib fractures. BJA Educ 20(10):332–340 33456914 10.1016/j.bjae.2020.06.001 PMC7807920

[91] O’Connell KM, Patel KV, Powelson E, Robinson BRH, Boyle K, Peschman J et al (2021) Use of regional analgesia and risk of delirium in older adults with multiple rib fractures: An Eastern Association for the Surgery of Trauma multicenter study. The Journal of Trauma and Acute Care Surgery 91(2):265–271 33938510 10.1097/TA.0000000000003258 PMC9704032

[92] Schweickert WD, Pohlman MC, Pohlman AS, Nigos C, Pawlik AJ, Esbrook CL et al (2009) Early physical and occupational therapy in mechanically ventilated, critically ill patients: a randomised controlled trial. Lancet 373(9678):1874–1882 19446324 10.1016/S0140-6736(09)60658-9 PMC9906655

[93] Fowler AJ, Abbott TEF, Prowle J, Pearse RM (2019) Age of patients undergoing surgery. Br J Surg 106(8):1012–1018 31115918 10.1002/bjs.11148

[94] Swarbrick CJ, Williams K, Evans B, Blake HA, Poulton T, Nava S et al (2025) Characteristics of older patients undergoing surgery in the UK: SNAP-3, a snapshot observational study. Br J Anaesth 134(2):328–340 39765405 10.1016/j.bja.2024.11.024 PMC11775840

[95] Chalk F, Palmer A (2025) The impact of ageing and frailty on wound healing. Br J Nurs 34(20):S27-s34 41196764 10.12968/bjon.2025.0023

[96] Maaneb de Macedo KA, Scott J, Janeway MG (2025) The interface of trauma surgery and interventional radiology: the trauma surgeon’s perspective. Semin Intervent Radiol 42(4):447–458 41234340 10.1055/s-0045-1810014 PMC12606062

[97] NICE. Rehabilitation after traumatic injury 2022 [Available from: https://www.nice.org.uk/guidance/ng211/resources/rehabilitation-after-traumatic-injury-pdf-66143770162117.

[98] Arun B, Lewis SHM (2026) Frailty and deconditioning on the acute take. Clin Med (Lond) 26(2):100548 41500396 10.1016/j.clinme.2025.100548 PMC12861237

[99] Pandor A, Essat M, Sutton A, Fuller G, Reid S, Smith JE et al (2024) Cervical spine immobilisation following blunt trauma in pre-hospital and emergency care: A systematic review. PLoS ONE 19(4):e0302127 38662734 10.1371/journal.pone.0302127 PMC11045128

[100] Peck GE, Shipway DJH, Tsang K, Fertleman M (2018) Cervical spine immobilisation in the elderly: a literature review. Br J Neurosurg 32(3):286–290 29488398 10.1080/02688697.2018.1445828

[101] Baji P, Patel R, Judge A, Johansen A, Griffin J, Chesser T et al (2023) Organisational factors associated with hospital costs and patient mortality in the 365 days following hip fracture in England and Wales (REDUCE): a record-linkage cohort study. Lancet Healthy Longev 4(8):e386–e398 37442154 10.1016/S2666-7568(23)00086-7

[102] Fisher JM, Bates C, Banerjee J (2017) The growing challenge of major trauma in older people: a role for comprehensive geriatric assessment? Age Ageing. 10.1093/ageing/afx035 28338866

[103] Bretherton CP, Hirst R, Gacaferi H, Gower J, Exell L, Johnston S et al (2024) Research priorities for the management of major trauma: an international priority setting partnership with the James Lind Alliance. BMJ Open 14(5):e083450 38754886 10.1136/bmjopen-2023-083450 PMC11107451

[104] Alshibani A, Banerjee J, Lecky F, Coats TJ, Prest R, Mitchell Á et al (2020) A consensus building exercise to determine research priorities for silver trauma. BMC Emerg Med 20(1):63 32825810 10.1186/s12873-020-00357-4 PMC7441540

[105] Joseph B, Saljuqi AT, Phuong J, Shipper E, Braverman MA, Bixby PJ et al (2022) Developing a National Trauma Research Action Plan: results from the geriatric research gap Delphi survey. J Trauma Acute Care Surg 93(2):209–219 35393380 10.1097/TA.0000000000003626

